# Supplementary material for: Effects of exogenous EBR on the physiology of cold resistance and the expression of the VcCBF3 gene in blueberries during low-temperature stress
Source: PLoS One. 2025 Feb 13;20(2):e0313194. doi: 10.1371/journal.pone.0313194 (PMC11825034; doi:10.1371/journal.pone.0313194)
Supplement: S1 File — (PDF) [file pone.0313194.s001.pdf]

TRIzol™ Reagent

Catalog Numbers 15596026 and 15596018

Doc. Part No. 15596026.PPS **Pub. No.** MAN0001271 **Rev.** B.0

⚠ **WARNING!** Read the Safety Data Sheets (SDSs) and follow the handling instructions. Wear appropriate protective eyewear, clothing, and gloves. Safety Data Sheets (SDSs) are available from **thermofisher.com/support**.

Product description

Invitrogen™ TRIzol™ Reagent is a ready-to-use reagent, designed to isolate high quality total RNA (as well as DNA and proteins) from cell and tissue samples of human, animal, plant, yeast, or bacterial origin, within one hour. TRIzol™ Reagent is a monophasic solution of phenol, guanidine isothiocyanate, and other proprietary components which facilitate the isolation of a variety of RNA species of large or small molecular size. TRIzol™ Reagent maintains the integrity of the RNA due to highly effective inhibition of RNase activity while disrupting cells and dissolving cell components during sample homogenization. TRIzol™ Reagent allows for simultaneous processing of a large number of samples, and is an improvement to the single-step RNA isolation method developed by Chomcynski and Sacchi (Chomczynski and Sacchi, 1987).

TRIzol™ Reagent allows to perform sequential precipitation of RNA, DNA, and proteins from a single sample (Chomczynski, 1993). After homogenizing the sample with TRIzol™ Reagent, chloroform is added, and the homogenate is allowed to separate into a clear upper aqueous layer (containing RNA), an interphase, and a red lower organic layer (containing the DNA and proteins). RNA is precipitated from the aqueous layer with isopropanol. DNA is precipitated from the interphase/organic layer with ethanol. Protein is precipitated from the phenol-ethanol supernatant by isopropanol precipitation. The precipitated RNA, DNA, or protein is washed to remove impurities, and then resuspended for use in downstream applications.

- Isolated RNA can be used in RT-PCR, Northern Blot analysis, Dot Blot hybridization, poly(A)+ selection, in vitro translation, RNase protection assay, and molecular cloning.
- Isolated DNA can be used in PCR, Restriction Enzyme digestion, and Southern Blots.
- Isolated protein can be used for Western Blots, recovery of some enzymatic activity, and some immunoprecipitation.

For DNA isolation, see the *TRIzol™ Reagent (DNA isolation) User Guide* (Pub. No. MAN0016385).

TRIzol™ Reagent can also be used with Phasemaker™ Tubes (Cat. No. A33248) to isolate RNA. Phasemaker™ Tubes creates a solid barrier between the organic and aqueous phases of the TRIzol™ Reagent following sample homogenization which makes separation of phases easier. See the *TRIzol™ Reagent and Phasemaker™ Tubes Complete System User Guide* (Pub. No. MAN0016163) for the full protocol.

TRIzol™ Reagent can also be used with the PureLink™ RNA Mini Kit (Cat. No. 12183018A) which uses spin columns instead of ethanol precipitation to purify the RNA. For additional information, see the *PureLink™ RNA Mini Kit User Guide* (Pub. No. MAN0000406).

Contents and storage

Contents	Cat. No. 15596026 (100 reactions)	Cat. No. 15596018 (200 reactions)	Storage
TRIzol™ Reagent	100 mL	200 mL	15–30°C

Required materials not supplied

Unless otherwise indicated, all materials are available through **thermofisher.com**. MLS: Fisher Scientific (**fisherscientific.com**) or other major laboratory supplier.

Table 1 Materials required for all isolations

Item	Source
**Equipment**	
Centrifuge and rotor capable of reaching 12,000 × *g* and 4°C	MLS
**Tubes**	
Polypropylene microcentrifuge tubes	MLS
**Reagents**	
Chloroform	MLS

Table 2 Materials required for RNA isolation

Item	Source
**Equipment**	
Water bath or heat block at 55–60°C	MLS
**Reagents**	
Isopropanol	MLS
Ethanol, 75%	MLS
RNase-free water of 0.5% SDS	MLS
(Optional) RNase-free glycogen	MLS

Table 3 Materials required for protein isolation

Item	Source
**Equipment**	
(Optional) Dialysis membranes	MLS
**Reagents**	
Isopropanol	MLS
Ethanol, 100%	MLS
0.3 M Guanidine hydrochloride in 95% ethanol	MLS
1% SDS	MLS

Input sample requirements

IMPORTANT! Perform RNA isolation immediately after sample collection or quick-freeze samples immediately after collection and store at –80°C or in liquid nitrogen until RNA isolation.

Sample type	Starting material per 1 mL of TRIzol™ Reagent
Tissues[1]	50–100 mg of tissue
Cells grown in monolayer	1×10^5–1×10^7 cells grown in monolayer in a 3.5-cm culture dish (10 cm^2)
Cells grown in suspension	5–10 $\times 10^6$ cells from animal, plant, or yeast origin or 1 $\times 10^7$ cells of bacterial origin

[1] Fresh tissues or tissues stored in RNA*later*™ Stabilization Solution (Cat. No. AM7020).

For Research Use Only. Not for use in diagnostic procedures.

Procedural guidelines

- Perform all steps at room temperature (20–25°C) unless otherwise noted.
- Use cold TRIzol™ Reagent if the starting material contains high levels of RNase, such as spleen or pancreas samples.
- Use disposable, individually wrapped, sterile plastic ware and sterile, disposable RNase-free pipettes, pipette tips, and tubes.
- Wear disposable gloves while handling reagents and RNA samples to prevent RNase contamination from the surface of the skin; change gloves frequently, particularly as the protocol progresses from crude extracts to more purified materials.
- Always use proper microbiological aseptic techniques when working with RNA.
- Use RNase*Zap*™ RNase Decontamination Solution (Cat. no. AM9780) to remove RNase contamination from work surfaces and non-disposable items such as centrifuges and pipettes used during purification.
- Ensure that all materials that come into contact with TRIzol™ Reagent are compatible with phenol, guanidine isothiocyanate, and chloroform.

Lyse samples and separate phases

1. Lyse and homogenize samples in TRIzol™ Reagent according to your starting material.
   - **Tissues:**

     Add 1 mL of TRIzol™ Reagent per 50–100 mg of tissue to the sample and homogenize using a homogenizer.
   - **Cell grown in monolayer:**
     a. Remove growth media.
     b. Add 0.3–0.4 mL of TRIzol™ Reagent per 1 × 10^5—10^7 cells directly to the culture dish to lyse the cells.
     c. Pipet the lysate up and down several times to homogenize.
   - **Cells grown in suspension:**
     a. Collect the cells by centrifugation and discard the supernatant.
     b. Add 0.75 mL of TRIzol™ Reagent per 0.25 mL of sample (5–10 × 10^6 cells from animal, plant, or yeast origin or 1 ×10^7 cells of bacterial origin) to the pellet.

        Note: Do not wash cells before addition of TRIzol™ Reagent to avoid mRNA degradation.
     c. Pipet the lysate up and down several times to homogenize.

   Note: The sample volume should not exceed 10% of the volume of TRIzol™ Reagent used for lysis.

   STOPPING POINT Samples can be stored at 4°C overnight or at –20°C for up to a year.
2. *(Optional)* If samples have a high fat content, centrifuge the lysate for 5 minutes at 12,000 × *g* at 4–10°C, then transfer the clear supernatant to a new tube.
3. Incubate for 5 minutes to allow complete dissociation of the nucleoproteins complex.
4. Add 0.2 mL of chloroform per 1 mL of TRIzol™ Reagent used for lysis, securely cap the tube, then thoroughly mix by shaking.
5. Incubate for 2–3 minutes.
6. Centrifuge the sample for 15 minutes at 12,000 × *g* at 4°C.

   The mixture separates into a lower red phenol-chloroform, an interphase, and a colorless upper aqueous phase.
7. Transfer the aqueous phase containing the RNA to a new tube by angling the tube at 45° and pipetting the solution out.

   IMPORTANT! Avoid transferring any of the interphase or organic layer into the pipette when removing the aqueous phase.

Proceed directly to "Isolate RNA" on page 2.

To isolate DNA or protein, save the interphase and organic phase. See the *TRIzol™ Reagent (DNA isolation) User Guide* (Pub. No. MAN0016385) or see "Isolate proteins" on page 3 for detailed procedures. The organic phase can be stored at 4°C overnight.

Isolate RNA

1 Precipitate the RNA

a. *(Optional)* If the starting sample is small (<10^6 cells or <10 mg of tissue), add 5–10 μg of RNase-free glycogen as a carrier to the aqueous phase.

   Note: The glycogen is co-precipitated with the RNA, but does not interfere with subsequent applications.

b. Add 0.5 mL of isopropanol to the aqueous phase, per 1 mL of TRIzol™ Reagent used for lysis.
c. Incubate for 10 minutes at 4°C.
d. Centrifuge for 10 minutes at 12,000 × *g* at 4°C.

   Total RNA precipitate forms a white gel-like pellet at the bottom of the tube.
e. Discard the supernatant with a micropipettor.

2 Wash the RNA

a. Resuspend the pellet in 1 mL of 75% ethanol per 1 mL of TRIzol™ Reagent used for lysis.

   Note: The RNA can be stored in 75% ethanol for at least 1 year at –20°C, or at least 1 week at 4°C.
b. Vortex the sample briefly, then centrifuge for 5 minutes at 7500 × *g* at 4°C.
c. Discard the supernatant with a micropipettor.
d. Vacuum or air dry the RNA pellet for 5–10 minutes.

   IMPORTANT! Do not dry the pellet by vacuum centrifuge. Do not let the RNA pellet dry, to ensure total solubilization of the RNA. Partially dissolved RNA samples have an $A_{230/280}$ ratio <1.6.

3 Solubilize the RNA

a. Resuspend the pellet in 20–50 μL of RNase-free water, 0.1 mM EDTA, or 0.5% SDS solution by pipetting up and down.

   IMPORTANT! Do not dissolve the RNA in 0.5% SDS if the RNA is to be used in subsequent enzymatic reactions.
b. Incubate in a water bath or heat block set at 55–60°C for 10–15 minutes.

Proceed to downstream applications, or store the RNA at –70°C.

4 Determine the RNA yield

Determine the RNA yield using one of the following methods.

Method	Procedure
Absorbance<br>Absorbance at 260 nm provides total nucleic acid content, while absorbance at 280 nm determines sample purity. Since free nucleotides, RNA, ssDNA, and dsDNS absorb at 260 nm, they all contribute to the total absorbance of the sample.	1. Dilute sample in RNase-free water, then measure absorbance at 260 nm and 280 nm.<br>2. Calculate the RNA concentration using the formula A260 × dilution × 40 = µg RNA/mL.<br>3. Calculate the A260/A280 ratio.<br>A ratio of ~2 is considered pure.<br>RNA samples can be quantified by absorbance without prior dilution using the NanoDrop™ Spectophotometer. Refer to the instrument's instructions for more information.
Fluorescence<br>Fluorescence selectively measures intact RNA, but does not measure protein or other contaminant present in the sample	• Quantify RNA yield using the appropriate Qubit™ or Quant-iT™ RNA Assay Kit (Cat. Nos. Q32852, Q10210, Q33140, or Q10213).<br>Refer to the kit's instructions for more information.

Table 4 Typical RNA ($A_{260/280}$ of >1.8) yields from various starting materials

Starting material	Quantity	RNA yield
Epithelial cells	1 × 10^6 cells	8–15 µg
New tobacco leaf	—	73 µg
Fibroblasts	1 × 10^6 cells	5–7 µg
Skeletal muscles and brain	1 mg	1–1.5 µg
Placenta	1 mg	1–4 µg
Liver	1 mg	6–10 µg
Kidney	1 mg	3–4 µg

Isolate proteins

Isolate the proteins from the organic phase saved from “Isolate RNA“ on page 2 using either “Precipitate the proteins“ on page 3 or “Dialyse the proteins“ on page 4.

1 Precipitate the proteins

a. Remove any remaining aqueous phase overlying the interphase.
b. Add 0.3 mL of 100% ethanol per 1 mL of TRIzol™ Reagent used for lysis.
c. Cap the tube, mix by inverting the tube several times.
d. Incubate for 2–3 minutes.
e. Centrifuge for 5 minutes at 2000 × *g* at 4°C to pellet the DNA.
f. Transfer the phenol-ethanol supernatant to a new tube.
g. Add 1.5 mL of isopropanol to the phenol-ethanol supernatant per 1 mL of TRIzol™ Reagent used for lysis.
h. Incubate for 10 minutes.
i. Centrifuge for 10 minutes at 12,000 × *g* at 4°C to pellet the proteins.
j. Discard the supernatant with a micropipettor.

2 Wash the proteins

a. Prepare a wash solution consisting of 0.3 M guanidine hydrochloride in 95% ethanol.
b. Resuspend the pellet in 2 mL of wash solution per 1 mL of TRIzol™ Reagent used for lysis.
c. Incubate for 20 minutes.

   Note: The proteins can be stored in wash solution for at least 1 month at 4°C or for at least 1 year at –20°C.
d. Centrifuge for 5 minutes at 7500 × *g* at 4°C.
e. Discard the supernatant with a micropipettor.
f. Repeat step 2b–step 2e twice.
g. Add 2 mL of 100% ethanol, then mix by vortexing briefly.
h. Incubate for 20 minutes.
i. Centrifuge for 5 minutes at 7500 × *g* at 4°C.
j. Discard the supernatant with a micropipettor.
k. Air dry the protein pellet for 5–10 minutes.

IMPORTANT! Do not dry the pellet by vacuum centrifuge.

3 Solubilize the proteins

a. Resuspend the pellet in 200 µL of 1% SDS by pipetting up and down.

   Note: To ensure complete resuspension of the pellet, we recommend that you incubate the sample at 50°C in a water bath or heat block.
b. Centrifuge for 10 minutes at 10,000 × *g* at 4°C to remove insoluble materials.
c. Transfer the supernatant to a new tube.

Measure protein concentration by Bradford assay (SDS concentration mush be <0.1%), then proceed directly to downstream applications, or store the sample at –20°C.

Dialyse the proteins

1. Remove any remaining aqueous phase overlying the interphase.
2. Add 0.3 mL of 100% ethanol per 1 mL of TRIzol™ Reagent used for lysis.
3. Cap the tube, mix by inverting the tube several times.
4. Incubate for 2–3 minutes.
5. Centrifuge for 5 minutes at 2000 × *g* at 4°C to pellet the DNA.
6. Load the phenol-ethanol supernatant into the dialysis membrane.

   Note: The phenol-ethanol solution can dissolve some types of dialysis membranes (cellulose ester, for example). Test dialysis tubing with the membrane to assess compatibility before starting.
7. Dialyze the sample against 3 changes of 0.1% SDS at 4°C. Make the first change of solution after 16 hours, the second change 4 hours later (at 20 hours), and the final change 2 hours later (at 22 hours).

   Note: A SDS concentration of at least 0.1% is required to resolubilize the proteins from the pellet. If desired, the SDS can be diluted after solubilization.
8. Centrifuge the dialysate for 10 minutes at 10,000 × *g* at 4°C.
9. Transfer the supernatant containing the proteins to a new tube.
10. *(Optional)* Solubilize the pellet by adding 100 µL of 1% SDS and 100 µL of 8 M urea.

Proceed directly to downstream applications, or store the sample at –20°C.

Troubleshooting

Troubleshooting and FAQs

Visit our online FAQ database for tips and tricks for conducting your experiment, troubleshooting information, and FAQs. The online FAQ database is frequently updated to ensure accurate and thorough content.

- For troubleshooting information and FAQs for this product: **https://www.thermofisher.com/trizolfaqs**
- To browse the database and search using keywords: **thermofisher.com/faqs**

Limited product warranty

Life Technologies Corporation and/or its affiliate(s) warrant their products as set forth in the Life Technologies' General Terms and Conditions of Sale at **www.thermofisher.com/us/en/home/global/terms-and-conditions.html**. If you have any questions, please contact Life Technologies at **www.thermofisher.com/support**.

References

Chomczynski, P. 1993. *A reagent for the single-step simultaneous isolation of RNA, DNA and proteins from cell and tissue samples.* BioTechniques *15*, 532-537

Chomczynski, P., and Sacchi, N. 1987 *Single Step Method of RNA Isolation by Acid Guanidinium Thiocyanate-Phenol-Chloroform Extraction.* Anal. Biochem. *162*, 156-159

Hummon, A. B., Lim S. R., Difilippantonio, M. J., and Ried, T. 2007 *Isolation and solubilization of proteins after TRIzol® extraction of RNA and DNA from patient material following prolonged storage.* BioTechniques *42*, 467-472

Life Technologies Corporation | 5781 Van Allen Way | Carlsbad, CA 92008

For descriptions of symbols on product labels or product documents, go to **thermofisher.com/symbols-definition**.

The information in this guide is subject to change without notice.

DISCLAIMER: TO THE EXTENT ALLOWED BY LAW, THERMO FISHER SCIENTIFIC INC. AND/OR ITS AFFILIATE(S) WILL NOT BE LIABLE FOR SPECIAL, INCIDENTAL, INDIRECT, PUNITIVE, MULTIPLE, OR CONSEQUENTIAL DAMAGES IN CONNECTION WITH OR ARISING FROM THIS DOCUMENT, INCLUDING YOUR USE OF IT.

Revision history: Pub. No. MAN00001271

Revision	Date	Description
B.0	28 January 2020	• Moved troubleshooting topics to **thermofisher.com**.<br>• Added note to ensure that materials that come into contact with the Trizol Reagent are compatible.<br>• Updated step 4 of "Lyse samples and separate phases" on page 2 to include shaking the sample.
A.0	09 November 2016	Added references to Phasemaker™ Tubes .
—	13 December 2012	Baseline for revision.

Important Licensing Information: These products may be covered by one or more Limited Use Label Licenses. By use of these products, you accept the terms and conditions of all applicable Limited Use Label Licenses.

©2020 Thermo Fisher Scientific Inc. All rights reserved. All trademarks are the property of Thermo Fisher Scientific and its subsidiaries unless otherwise specified.
